# Supplementary material for: End Sequence Analysis Toolkit (ESAT) expands the extractable information from single-cell RNA-seq data
Source: Genome Res. 2016 Oct;26(10):1397–410. doi: 10.1101/gr.207902.116 (PMC5052061; doi:10.1101/gr.207902.116)
Supplement: Supplemental Material [file supp_gr.207902.116_Supp_Materials.tar.gz › ESAT-esat_v0.1/doc/html/classbroad_1_1pda_1_1seq_1_1fastq_1_1_fastq_sequence-members.html]

ESAT: Member List


ESAT


- Main Page
- Related Pages
- Packages
- Classes
- Files

- Class List
- Class Index
- Class Hierarchy
- Class Members

All Classes Namespaces Files Functions Variables Enumerator Pages

- broad
- pda
- seq
- fastq
- FastqSequence

broad.pda.seq.fastq.FastqSequence Member List

This is the complete list of members for broad.pda.seq.fastq.FastqSequence, including all inherited members.

|  |  |  |
| --- | --- | --- |
| FastqSequence(String name, String sequence, String description, String quality) | broad.pda.seq.fastq.FastqSequence |  |
| getDescription() | broad.pda.seq.fastq.FastqSequence |  |
| getFirstNBPs(int n) | broad.pda.seq.fastq.FastqSequence |  |
| getLength() | broad.pda.seq.fastq.FastqSequence |  |
| getName() | broad.pda.seq.fastq.FastqSequence |  |
| getSequence() | broad.pda.seq.fastq.FastqSequence |  |
| isPartialPolyA(int polyN) | broad.pda.seq.fastq.FastqSequence |  |
| isPolyA() | broad.pda.seq.fastq.FastqSequence |  |
| removeAtSymbolFromName() | broad.pda.seq.fastq.FastqSequence |  |
| setDescription(String descr) | broad.pda.seq.fastq.FastqSequence |  |
| setName(String name) | broad.pda.seq.fastq.FastqSequence |  |
| toFasta() | broad.pda.seq.fastq.FastqSequence |  |
| toFasta(int readNum) | broad.pda.seq.fastq.FastqSequence |  |
| toFastq() | broad.pda.seq.fastq.FastqSequence |  |
| toString() | broad.pda.seq.fastq.FastqSequence |  |
| trimBeginning(char letter) | broad.pda.seq.fastq.FastqSequence |  |
| trimEndBases(int numOfBasesToTrim) | broad.pda.seq.fastq.FastqSequence |  |
| trimEnds(char letter) | broad.pda.seq.fastq.FastqSequence |  |
| trimFirstNBPs(int n) | broad.pda.seq.fastq.FastqSequence |  |
| trimLinker(String linkerSeq) | broad.pda.seq.fastq.FastqSequence |  |
| trimPolyA() | broad.pda.seq.fastq.FastqSequence |  |
| trimStartBases(int numOfBasesToTrim) | broad.pda.seq.fastq.FastqSequence |  |
| write(BufferedWriter bw) | broad.pda.seq.fastq.FastqSequence |  |


---

Generated on Fri Aug 15 2014 15:11:02 for ESAT by  

 1.8.7
